# Evaluation of urban green space plant landscape quality in Zhengzhou city using the AHP-SBE method

**DOI:** 10.1371/journal.pone.0329119

**Published:** 2025-09-26

**Authors:** Lingling Zhang, Yuan Gao, Qiutan Ren, Shidong Ge, Bingquan Wang, Chong Du, Yiping Liu, Dezheng Kong

**Affiliations:** 1 College of Landscape Architecture and Art, Henan Agricultural University, Zhengzhou, China; 2 Department of Art and Design, Zhengzhou Business University, Gongyi, China; 3 MOE Key Laboratory of Tibetan Plateau Land Surface Processes and Ecological Conservation, and School of the Geographical Science, Qinghai Normal University, Xining, China; 4 SuperMap Software Co., Ltd, Zhengzhou, China; Shahid Beheshti University, IRAN, ISLAMIC REPUBLIC OF

## Abstract

Urban green space plays a key role in urban ecosystems, and the quality of the landscape directly affects its ecological, aesthetic, and social functions. On the basis of field survey data from 349 sample sites in Zhengzhou City, this study constructed a comprehensive evaluation index system for plantscape and systematically evaluated 40 representative plantscape units using the hierarchical analysis method (AHP) and the scenic beauty estimation method (SBE). The results indicated that: (1) In the analysis of plant diversity, the diversity indices for trees, shrubs, and herbs in park green spaces were higher than those observed in other types of green spaces, with overall species richness reaching its highest level. (2) The results of the AHP comprehensive evaluation revealed that 10% of the samples were classified as high-quality (CEI ≥ 8 points), 57.5% as medium-to-high quality (6 ≤ CEI < 8 points), and 32.5% as medium or below (CEI < 6 points). The mean comprehensive scores across different green space types were ranked as follows: park green space> ancillary green space> protective green space> regional green space> plaza land. (3) The SBE evaluation results showed that only 40% of the samples achieved standardized scores above zero, while 60% scored below zero, indicating that the overall aesthetic quality of the planted landscapes was low and required optimization and improvement. (4) Spearman’s correlation analysis revealed that the AHP and SBE methods were highly consistent in terms of the ranking of the plant landscape units, and there was a significant positive correlation. This study combines quantitative evaluation and perceptual analysis methods to systematically reveal the characteristics and differences of plant escape among different types of green space in Zhengzhou City, which provides a theoretical basis and practical reference for the optimal design of plant escape in urban green spaces and the high-quality development of these spaces.

## Introduction

Cities are areas where high-intensity human activities coexist with rich biodiversity [1], and urban green spaces, as vital components of the urban fabric, fulfill multiple ecological and social functions, including absorbing harmful gases, providing isolation buffers, and offering resting areas. With the progression of urbanization, the development of urban green spaces not only enhances ecological benefits—such as climate regulation, mitigation of waterlogging, and maintenance of biodiversity—but also delivers recreational and cultural experiences for citizens [2]. Plant diversity, as the core element of green space ecosystems, directly influences both the ecological functionality and the landscape value of green spaces [3]. Consequently, the scientific and rational planning and management of plant resources are essential for optimizing the urban ecological environment and improving landscape quality. Plant landscape evaluation, as an effective assessment tool, is widely employed in the planning and management of urban green spaces. Commonly used plant landscape evaluation methods include the hierarchical analysis method (AHP) [4], the scenic beauty estimation method (SBE) [5]and the semantic difference method (SD) [6,7]. The AHP method integrates qualitative and quantitative data, scientifically defines index weights, and is well-suited for multidimensional comprehensive evaluations. In contrast, the SBE method emphasizes subjective aesthetic perceptions of the landscape and experiential effects. Together, these methods complement each other and contribute to the development of a more comprehensive plant landscape evaluation system. By applying both approaches, the strengths and weaknesses of green spaces can be identified, providing a scientific basis for optimizing plant landscapes and enhancing their ecological benefits and social functions.

In recent years, scholars at home and abroad have conducted many studies on the evaluation of planted landscapes, revealing their important role in urban planning and ecological environmental protection. Hu X et al. [8] applied the Scenic Beauty Estimation (SBE) method to systematically evaluate the plant communities of Tongjian Lake in Hangzhou, proposing strategies to enhance the landscape quality of urban waterfront greenways across dimensions such as ecology, aesthetics, and public services. Peng X et al. [9] employed the AHP-SBE method to assess the landscape quality of six pocket parks in Dadukou District, Chongqing City, recommending the enhancement of plant diversity, optimization of color design to improve visual attractiveness, and the adoption of sustainable, low-maintenance strategies. Y. Shi et al. [10] integrated the AHP and SBE methods to comprehensively evaluate the botanical landscape units of a park in Kunming City, subsequently proposing optimized design strategies. International research has similarly emphasized the assessment of plantscapes in urban green spaces; for example, Baumgardner D. [11] quantitatively analyzed the plant ecosystems of Mexican urban national parks using the UFORE model. Ali Ozbilen et al. [12] explored the influence of plant community assemblages on public perception through the semantic difference (SD) method. Yan H et al. [13] used the AHP and SBE methods to reveal the correlation between several water quality indicators and landscape aesthetics indices of wetlands, which provides a reference for the practical design and maintenance management of urban landscapes.

Despite the positive advancements of existing studies, most have concentrated on the eastern coastal regions and major urban agglomerations in China [14–16], with a notable lack of systematic research on urban green space plant landscapes in central China, particularly in Zhengzhou. As the core city of the Central Plains urban agglomeration, Zhengzhou is densely populated and has undergone a dramatic urbanization process, making it a representative area for studying the impacts of urbanization on plant diversity [17]. In recent years, rapid urbanization has significantly affected the area, quality, and plant diversity of green spaces in Zhengzhou, leading to issues such as homogenization of plant configurations, degradation of native species, and invasion by exotic species, all of which have hindered improvements in urban ecological function and landscape quality [18]. Consequently, there is an urgent need to assess plant landscape quality using scientific methods, accurately diagnose existing issues, and propose targeted optimization strategies. Furthermore, challenges such as reliance on empirical judgments, insufficient quantification, and unstandardized construction of indicator systems persist in plant landscape evaluations, limiting their scientific rigor and practical applicability. To address these issues, this study introduces the AHP-SBE method, leveraging its strengths in systematically decomposing problems, scientifically determining indicator weights, and integrating both subjective and objective information, to construct a scientific, rational, and multidimensional plant landscape evaluation system [10], aiming to enhance the systematicity, scientific validity, and practical guidance value of the evaluation.

Therefore, this study takes the urban green space in Zhengzhou City as the object and systematically evaluates the quality of the plant landscape from the aspects of landscape composition, structural characteristics, aesthetic effects and ornamental psychology through field research and comprehensive application of the landscape evaluation method. The objectives of this study are: (1) to reveal the current status and characteristics of plant diversity across different types of green spaces in Zhengzhou City; (2) to explore the applicability and distinctive features of the AHP and SBE methods in the evaluation of plant landscapes; and (3) to compare the differences and consistencies between the evaluation results of the two methods and validate their complementary advantages. Based on the findings, specific strategies and recommendations for optimizing plant landscapes in Zhengzhou are proposed, with the aim of providing practical support for enhancing plant diversity in urban green spaces and improving the ecological environment and landscape quality. Furthermore, this study aspires to offer theoretical references and practical experiences for plant landscape evaluation and green space optimization in other cities in the central region and across China.

## Materials and methods

The dataset utilized in this study was derived from questionnaires and did not involve human clinical trials or the collection of sensitive personal information. All research procedures adhered to established ethical standards. The study protocol was approved by the Ethics Committee of the College of Landscape Architecture and Art, Henan Agricultural University. Prior to participation, all respondents were informed of the study’s purpose, content, and the intended use of the data, and voluntarily provided written informed consent. Participants’ information was kept strictly confidential and was used solely for academic research purposes.

### Study area

**Fig 1:**
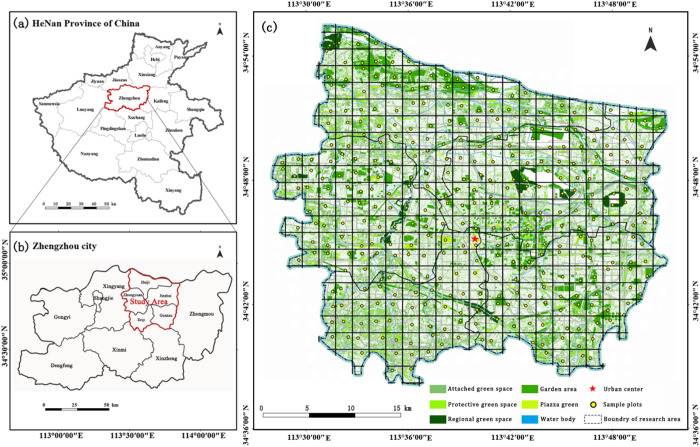
Study area and sample plot distribution. The map data for Henan Province, Zhengzhou City, and Zhengzhou’s main urban area are sourced from the National Geographic Information Public Service Platform (Tianditu) website (https://cloudcenter.tianditu.gov.cn/administrativeDivision), provided by the National Basic Geographic Information Center. As the map resources offered by these two websites are freely available for academic use, our research does not need to supply a copyright notice.

Zhengzhou is located in the north-central part of Henan Province, at the heart of the Central Plains region, and is known as the “Center of Nine Provinces,” with geographic coordinates ranging from 112°42′E to 114°14′E and 34°16′N to 34°58′N (Fig 1). The regional topography generally exhibits a pattern of higher elevations in the southwest and lower elevations in the northeast. Zhengzhou experiences a warm-temperate, semi-humid continental monsoon climate, characterized by four distinct seasons, an average annual temperature of about 14.3 °C, and annual precipitation of about 640–740 mm, predominantly concentrated in the summer months [19]. The diverse landforms, including mountains, hills, and plains, combined with pronounced seasonal variation, have fostered a rich array of plant resources in the region. According to the Flora of Zhengzhou, the city hosts 181 families, 941 genera, and 2,302 species, subspecies, and varieties of vascular plants [20]. Among them are numerous rare, endangered, and endemic species, such as the nationally protected plants Metasequoia glyptostroboides, Ginkgo biloba, Glycine soja, and Pinus bungeana. These species not only have important ecological and genetic value but also have high requirements for urban plant diversity conservation. The rich plant resources provide a solid foundation and a broad research space for this study.

The urban green space system in Zhengzhou City primarily comprises park green spaces, protective green spaces, ancillary green spaces, plazas, and regional green spaces [21], forming a relatively complete and interconnected green space network. According to 2022 statistics, the green space rate within the built-up area of Zhengzhou reached 36.8% [22]. Among these, parks and green spaces are widely distributed across various functional zones of the city, characterized by high landscape quality and strong public service functions. Protective green spaces are arranged along rivers and urban peripheries, playing critical roles in ecological isolation and environmental regulation. Ancillary green spaces, associated with residential communities, schools, and hospitals, contribute to enhancing the urban living environment. Plazas, mainly concentrated in the city center, serve both urban beautification and public activity functions. Regional green spaces are located on the outskirts of the city, emphasizing ecological conservation and nature protection [23]. The diverse green space structure of Zhengzhou not only reflects the characteristics of rapid urban expansion and ecological construction but also provides a rich and diverse sample base for the study of plant diversity and landscape patterns.

### Sampling design and data collection

This study employed a grid-based random sampling method [24], utilizing Landsat 8 OLI remote sensing imagery and ArcGIS technology to delineate the administrative boundaries of Zhengzhou’s main urban area and extract urban green space coverage data. The study area was divided into 1.5 km × 1.5 km grids, with the number of samples determined according to the area occupied by different types of green spaces. Within each grid, 1–3 large green patches were randomly selected, resulting in a total of 349 sampling plots (Fig 1). Each plot covered an area of 400 m^2^, with a preference for a 20 m × 20 m square layout. Where terrain or other conditions constrained this arrangement, plots were adjusted to rectangular shapes of equivalent area. For sites lacking vegetation or those that were inaccessible, a similar replacement site within a 500 m radius was selected [25,26].

From May to October 2023, a six-month field investigation was conducted, encompassing vegetation stratification, plant community recording, and plant landscape photography. Vegetation stratification categorizes plants into three layers on the basis of height: the tree layer (>3 m), shrub layer (0.6–3 m), and ground cover layer (<0.6 m). Data collection included plant names, counts, growth conditions, species composition, density, diameter at breast height (DBH), height, and crown width. On clear weather days, representative plant landscape units within each plot were photographed to ensure the acquisition of high-quality images for subsequent analysis.

To study the influence of different green space types on plant landscape quality, 8 typical sample plots were selected from each of the 5 green space types, totaling 40 typical sample plots. The selection of typical plots was based on (1) spatial distribution uniformity, covering different green space types in the study area; (2) diversity of plant compositions, prioritizing the selection of plots with rich species, complete tree-shrub-grass hierarchies, and moderate environmental impacts; and (3) avoidance of severely degraded or strongly disturbed areas to ensure the universality of the results. Within each typical sample plot, representative landscape units were further selected to construct a screening system based on aesthetic value (B1), ecological value (B2), and social value (B3) [18]. The application of these core criteria ensures the scientific rigor and representativeness of the landscape units within the multidimensional evaluation system, providing foundational data and analysis samples for the AHP and SBE evaluation methods.

### Calculation of plant diversity indices

Species richness refers to the number of species within a given area. In this study, the Margalef species richness index (R) was employed for its calculation. Species diversity indices offer a comprehensive reflection of species quantity, structural composition, and distribution evenness. Commonly used indices include the Shannon-Wiener species diversity index (H), Simpson species dominance index (D) and Pielouspecies evenness index (J) [27–29]. On the basis of the vascular plant community system, this study utilized MATLAB 2019b software to calculate various diversity indices for 349 sampling plots. The relevant formulas are as follows:

*Margalef* species richness index


$$R=\frac{S-1}{l_{n}N}$$ (1)

*Shannon-Wiener* species diversity index


$$H=-\sum {\mathrm{\backslash nolimits}}_{i=1}^{S}(P_{i})(l_{n}p_{i})$$ (2)

*Simpson* species dominance index


$$D=1-\sum {\mathrm{\backslash nolimits}}_{i=1}^{S}(P_{i}{)}^{2}$$ (3)

*Pielou* species evenness index


$$J=\frac{H}{l_{n}S}$$ (4)

where  N is the total number of individuals, S is the number of species in the plot, $P_{i\;}$ is the proportion of individuals of species i relative to the total number of species in the plots, $P_{i}$=$N_{i}$/N, Ni is the number of individuals of speciesi, and $l_{n}$ is the natural logarithm with a base of 2.72 [30].

### AHP hierarchical analysis method

#### Selection and quantification of evaluation model indicators.

Following the principles of scientificity, objectivity, representativeness, and localization, this study constructed a comprehensive evaluation model of Zhengzhou’s urban green land plantscape with aesthetic value (B1), ecological value (B2), and social value (B3) as the core criteria. The members of the expert group for the AHP method were selected based on the principles of professionalism and representativeness. The group consisted of 15 individuals with relevant expertise, including 5 professors specializing in landscape architecture, 4 associate professors in urban ecology and environmental science, and 6 senior urban green space planning designers with more than 10 years of industry experience. All experts possessed professional backgrounds related to plant landscape evaluation, urban green space planning, or ecological protection, enabling them to provide scientific and rational judgment support for the weighting of the indicator system. Experts were recruited through targeted invitations to ensure coverage across different fields of research, design, and practice. Following multiple rounds of expert panel discussions and questionnaire feedback conducted between May and July 2024, a comprehensive assessment system comprising 18 indicators was finalized. Referring to the related study of Mo L et al. [5], quantitative and qualitative indicators were quantified separately. Quantitative indicators were calculated directly according to established interpretation rules, while qualitative indicators were obtained through expert questionnaire scores, categorized as excellent (8–10 points), good (6–8 points), moderate (4–6 points), poor (2–4 points), and very poor (0–2 points) [6], providing a clear framework for the scientific application of the evaluation system (details provided in Table 1, Appendix 1 in S1 File). The comprehensive evaluation model constructed in this study offers a solid theoretical foundation and practical methodology for the systematic evaluation of urban green space plantscapes in Zhengzhou City, supported by a scientific index system, standardized scoring criteria, and a rigorous expert selection process.

**Table 1:** Evaluation index and specific standard of of the plant landscape of urban green space in Zhengzhou.

Target layer	Standardized layer	Factor level	Evaluation levels and scores				
			Excellent(8–10)	Good (6–8)	Average (4–6)	Poor (2–4)	Very poor (0–2)
Comprehensive Evaluation of Zhengzhou Urban Greenland Plantscape A	Aesthetic value B1	Green visibilityC1	≥60%	45%-60%	30%-45%	15%-30%	≤15%
		Diversity of ornamental characteristics C2	Rich ornamental characteristics, with flowers, foliage, fruit, shape plants	With flowers, foliage, fruit and shape of plants in three of these categories	Two of the categories of plants with flowers, foliage, fruits, and shapes	Only one of the categories of flowering, foliage, fruiting, and formative plants	No flower, foliage, fruit, shape plants
		Hierarchical richness of communities C3	Very rich levels, ≥ 30 species of trees, shrubs and grasses, distinctive features	More abundant layers, with 21–29 species of trees, shrubs and grasses, and more distinctive features	General level, 11–20 species of trees, shrubs and grasses, general characteristics	Poor hierarchy, with 6–10 species of trees, shrubs and grasses, with no obvious characteristics	No hierarchy, ≤ 5 species of trees, shrubs and grasses, no special features at all
		Seasonal phase and color change C4	The landscape performance of each season is distinct, with strong visual appeal, rich color and natural transition	At least three seasons have unique landscape features, with good color matching and certain transition levels	Outstanding landscape performance only in one or two seasons, with reasonable color matching and bland visual effect of seasonal change	Most of the time the landscape performance is bland, lack of seasonal characteristics, single color, lack of change and sense of hierarchy, low visual appeal	Weak or the same landscape characteristics in each season, monotonous color, no transition level, poor sensory experience
		Space creation and scale coordination C5	Plant planting is very regular, space creation is very rich, with different types of space, and the scale is very coordinated	Spatial creation is reasonable, with one or two different spatial types, and the overall scale is comfortable	Spatial creation is not outstanding, only open or closed space, but the scale is coordinated as a whole	Overall bland, no obvious spatial characteristics, no obvious sense of scale	Chaotic planting, spatial disorganization, and very incongruous scale
		Harmony between plant community and surrounding environmentC6	The plant landscape is perfectly integrated with the surrounding environment, and the landscape is very harmonious	The plant landscape is similar to the surrounding environment and can reflect each other	The plant landscape is basically in harmony with the surrounding environment, and there is partial connection	The plant landscape has no obvious connection with the surrounding environment, but it is not abrupt	The plant landscape is isolated, without any connection with the outside world and very abrupt
	Ecological value B2	Plant species diversityC7	Plant species ≥ 15	10-14 plant species	6-10 plant species	3-5 plant species	Plant species ≤ 2
		Life-type diversity C8	With 8 different plant life types	6 out of 8 different plant life types	Has 4 of the 8 different plant life types	Having 2 of the 8 different plant life types	Not having any plant or any plant life type
		Plant health status C9	Vigorous growth, full plant shape, no pests and diseases, glossy and bright leaves, normal development of flowers and fruits	Overall growth is good, with occasional diseases, a little wilting, and normal flowering and fruiting	Normal but slightly sparse growth, mildly infested with pests and diseases, slightly dull, partially wilted foliage	Poor growth, noticeably sparse or wilted, more pronounced pests and diseases, generalized wilting of foliage, widespread loss of foliage, and impeded flowering and fruiting	Plants in serious decline or near death, with total wilting of foliage, no normal flowering and fruiting, and loss of plant vigor
		Environmental amelioration capacity C10	Good improvement effect on the environment, and strong improvement ability, can achieve sustainable development	The plants in the community as a whole have the ability to conserve soil and water and absorb harmful gases	Only a single or individual plant has the ability to improve the environment	Does not show outstanding environmental amelioration, but does not cause pollution	The plant community does not have any ability to improve the environment and is prone to pollution
		Capability of resisting C11	Strong adaptability to various adverse environmental conditions, fast self-repair and excellent tolerance	Able to adapt to most of the environmental stresses, able to repair themselves quickly, and with good tolerance	Tolerant of adverse environmental conditions, slow to recover, show moderate tolerance	Showing low tolerance to adverse environmental conditions, poor resistance to pests and diseases, insufficient tolerance	Little or no ability to adapt to adverse environmental conditions, difficult to recover from adversity, very low tolerance and adaptability
	Social value B3	Native Plant Ornamental Characteristics C12	The proportion of native plants is ≥ 80%, with strong visual attraction, significant landscape highlights in all seasons, and can be used as the core plants or landscape focal points	The proportion of native plants is between 60% and 80%, with a certain degree of ornamental, more obvious seasonal changes, and forming a good match with other plants	The proportion of native plants ranges from 40% to 60%, with medium ornamental value, insignificant seasonal changes, and need to be paired with other plants to emphasize their beauty	The proportion of native plants ranges from 20% to 40%, with low ornamental value, weak seasonal changes, and easy to be covered by other plants	Proportion of native plants ≤ 20%, poor visual performance, lack of seasonal changes, no sense of presence in the green space
		Cultural Symbol Connotation C13	The plants have deep historical and cultural deposits, are highly compatible with local culture, and the cultural connotation is widely disseminated	Plants have a certain cultural background or symbolic significance, a good fit with the local culture, and a certain degree of visibility of cultural connotations	The cultural significance of the plants is relatively common, generally associated with local culture, and the cultural connotation is less influential	The plants basically do not have significant cultural background or symbolic meaning, and have low relevance to the local culture and very low cultural influence	The plants do not have any cultural background or symbolic significance, do not fit in with the local culture, and have no cultural influence at all
		Conservation and Utilization of Old and Valuable Trees C14	There are many old and famous trees in the community, which are well protected and fully utilized, giving full play to their value	There are some old and famous trees in the community, and the existing trees are well protected	There are a small number of old and valuable trees in the community, but they are not completely protected or only individual valuable trees are protected and utilized	There are no old and famous trees in the community, and the existing trees have suffered some damage	There are no old and valuable trees in the community, and the existing trees have suffered serious damage
		Stayability C15	The landscape features are obvious, with space and facilities for viewing, strong ability to attract tourists to stop, and the tourists’ stay time is ≥ 8 min	Landscape with characteristics, with the conditions for tourists to stay for a short period of time, and the tourists’ stay time is 6–7 min	The landscape has certain characteristics, but there is not enough space or facilities for visitors to stay for 4–5 min	The landscape is relatively boring, only a few tourists are willing to stop and watch, and the tourists’ stay time is 2–3 min	The landscape is monotonous and does not have space and facilities to stay and watch, not attracting tourists, and the tourists’ stay time is ≤ 1 min
		Completeness of Supporting Facilities C16	Complete variety, sufficient quantity, strong functionality, reasonable layout and good maintenance	A complete range of species, sufficient quantity, strong functionality, reasonable layout and good maintenance	General species, general quantity, general functionality, general layout, general maintenance	Lack of species, insufficient number, poor functionality, irrational layout and poor maintenance	Scarcity of species, very few in number, poor functionality, chaotic layout, poor maintenance
		Safety C17	Stable community structure, non-toxic, no thorns, no plant tilt, no invasive plants	Stable community structure, individual plants with poisonous thorns, no obvious inclination, fewer exotic plants	General community structure, some plants are poisonous and have thorns, some trees are leaning, a few exotic plants	Unstable community structure, obviously poisonous with thorns, a large number of dead branches and leaning, a high proportion of invasive plants	The community structure is seriously unstable, a large number of plants have thorns and poisonous, there are a lot of dead branches and broken trees, and a large number of invasive plants
		Landscape Comfort C18	Rich plant species, open space, appropriate lighting, strong ornamental properties, and long residence time	More plant species, more open space, more suitable light, better visual attraction	Average plant species and levels, fair use of space, poor lighting, not attractive enough	Single plant species, poor spatial design, sun exposure, short residence time	Lack of plant species, chaotic spatial design, too much light, unwilling to stay

### Determination of weight values and consistency check

On the basis of the comprehensive evaluation model, the mean judgment matrix was constructed by combining the arithmetic mean values of different expert ratings. The sum-product method is used to calculate the weight values, and the consistency is tested. The steps and formulas are as follows:

#### (1) Hierarchical single ranking and consistency check.

① Calculate the product of the indicators:

$$Mi=\prod_{j=1}^{n}aij(i,j=1,2,3,\cdots \cdots ,n)$$ (5)

② Finding the mean by squaring n times:

$$\overset{―}{Wi}=\sqrt[{n}]{Mi}$$ (6)

③ Normalization process:

$$W_{i}=\frac{\overset{―}{W_{i}}}{\sum_{j=i}^{n}\overset{―}{W_{j}}}$$ (7)

④ Calculate the maximum characteristic root:

$$\lambda_{\max}=\frac{\text{1}}{n}\sum_{i=1}^{n}\frac{(AW)i}{Wi}$$ (8)

where *A* is the judgment matrix, *n* is the order of the judgment matrix, and ${\left(AW\right)}_{i\;}\;$ is the i component of the vector AW [10,31].

⑤ Consistency ratio calculation:

$$C.R.=\frac{C.I.}{R.I.}$$ (9)

where  C.I. is the consistency index and where R.I. is the mean random consistency index.

⑥ Coherence conditions:

$$C.I.=\frac{\lambda_{\max}-n}{n-1}$$ (10)

where *n* is the order of the judgment matrix and where $\lambda_{\max}$ is the maximum eigenvalue of the judgment matrix.

Finally, the value of the average random consistency index R.I. was referenced from the corresponding values in Table 2. By calculating the consistency ratio .R., the judgment matrix was considered to have passed the consistency check if C.R.<0.1; otherwise, adjustments to the matrix were required.

**Table 2:** Reference table of the average random consistency index of *R.I.*

Order	1	2	3	4	5	6	7	8	9	10
*R.I.*	0	0	0.52	0.89	1.12	1.26	1.36	1.41	1.46	1.49

The consistency ratio value  C.R. is calculated via Equations 5–10. IfC.R.<0.1, the judgment matrix is consistent; ifC.R.≥0.1, the judgment matrix fails the consistency check and must be adjusted and recalculated [18,32].

#### (2) Hierarchical total ordering and consistency test.

For the multi-level structure of the factor layer, this study conducted a total ranking and consistency check. The total ranking calculates the comprehensive weights of the factor layer (final level) relative to the target layer (highest level), with weight values derived through step-by-step transmission. The verification formula is as follows:


$$C.R.=\frac{\sum_{i=1}^{n}C.I.iWi}{\sum_{i=1}^{n}R.I.iWi}$$ (11)

where n represents the order of the judgment matrix, ${\;C.I.}_{i\;}$ is the consistency index for hierarchical single ranking, ${\;R.I.}_{i\;}$ is the average random consistency index, and ${\;W}_{i\;}$ is the total weight of the criterion layer (layer B). If C.R. <0.1, the total ranking passes the consistency check, indicating that the judgment matrix is consistent; if C.R. ≥0.1, the matrix lacks consistency and requires adjustment and recalculation [33].

#### (3) Total weight of the evaluation index system.

In accordance with the above methods and steps, the total weights of the evaluation index system are calculated (Tables 3–6 in Appendix 1 in S1 File for details). The results show that the consistency test results of hierarchical single sorting and total sorting meet the requirements and that all judgment matrices pass the consistency test.

On the basis of Table 3–6 and combined with Equation 11, the total weight table for the comprehensive evaluation of the plant landscape in the urban green space in Zhengzhou City was calculated. The consistency ratio *C.R.* value of hierarchical total sorting is 0.024, which is less than 0.1, indicating that the hierarchical total sorting passes the consistency test (Table 7).

**Table 3:** Mean value matrix and weight of criterion layer for comprehensive evaluation of urban green space plant landscape in Zhengzhou.

Matrix 1	Aesthetic value	Ecological value	Social value	Weighted value
Aesthetic value	1.00	0.33	2.00	0.24
Ecological value	3.00	1.00	4.00	0.62
Social value	0.50	0.25	1.00	0.14

In this matrix, *λmax* = 3.018, *C.I.* = 0.009, *R.I.* = 0.520, *C.R.* = 0.018 < 0.1, and the consistency test passes.

**Table 4:** ‘Aesthetic value’ factor layer mean value matrix and weight.

Matrix 2	Green visibility	Diversity of Ornamental Characteristics	Hierarchical richness of communities	Seasonal phase and color change	Space creation and scale coordination	Harmony between plant community and surrounding environment	weighted value
Green visibility	1.00	0.33	0.20	0.25	0.50	0.50	0.05
Diversity of ornamental characteristics	3.00	1.00	0.33	0.50	2.00	2.00	0.15
Hierarchical richness of communities	5.00	3.00	1.00	2.00	5.00	5.00	0.40
Seasonal phase and color change	4.00	2.00	0.50	1.00	2.00	2.00	0.21
Space creation and scale coordination	2.00	0.50	0.20	0.50	1.00	1.00	0.09
Harmony between plant community and surrounding environment	2.00	0.50	0.20	0.50	1.00	1.00	0.09

In this matrix, *λmax* = 6.086, *C.I.* = 0.017, *R.I.* = 1.260, *C.R.* = 0.014 < 0.1, and the consistency test was passed.

**Table 5:** ‘Ecological value’ factor layer mean value matrix and weight.

Matrix 3	Plant species diversity	Life-type diversity	Plant healthstatus	Environmental amelioration capacity	Capability of resisting	weighted value
Plant species diversity	1.00	4.00	3.00	2.00	5.00	0.41
Life-type diversity	0.25	1.00	0.50	0.33	2.00	0.10
Plant health status	0.33	2.00	1.00	0.33	3.00	0.15
Environmental amelioration capacity	0.50	3.00	3.00	1.00	4.00	0.28
Capability of resisting	0.20	0.50	0.33	0.25	1.00	0.06

In this matrix, *λmax* = 5.121, *C.I.* = 0.030, *R.I.* = 1.120, *C.R.* = 0.027 was < 0.1, and the consistency test was passed.

**Table 6:** ‘Social value’ factor layer mean value matrix and weight.

Matrix 4	Native Plant Ornamental Characteristics	Cultural Symbol Connotation	Conservation and Utilization of Old and Valuable Trees	Stayability	Completeness of Supporting Facilities	Safety	Landscape comfort	weighted value
Native Plant Ornamental Characteristics	1.00	3.00	2.00	5.00	0.50	0.25	4.00	0.15
Cultural Symbol Connotation	0.33	1.00	0.50	3.00	0.25	0.20	2.00	0.07
Conservation and Utilization of Old and Valuable Trees	0.50	2.00	1.00	4.00	0.33	0.25	3.00	0.11
Stayability	0.20	0.33	0.25	1.00	0.17	0.14	0.50	0.03
Completeness of Supporting Facilities	2.00	4.00	3.00	6.00	1.00	0.50	4.00	0.23
Safety	4.00	5.00	4.00	7.00	2.00	1.00	6.00	0.36
Landscape Comfort	0.25	0.50	0.33	2.00	0.25	0.17	1.00	0.05

In this matrix, *λmax* = 7.242, *C.I.* = 0.040, *R.I.* = 1.360, *C.R.* = 0.030 was < 0.1, and the consistency test was passed.

**Table 7:** Weights summary of the comprehensive evaluation of urban green space plant landscapes in Zhengzhou.

Goal level	Criteria Layer	Criteria layer weights	Factor Layers	Factor LayerWeights	Total Weights
Comprehensive evaluation of plant landscape of urban green space in Zhengzhou city A	Aesthetic value B1	0.24	Green visibility C1	0.05	0.01
			Diversity of ornamental characteristics C2	0.15	0.04
			Hierarchical richness of communities C3	0.40	0.10
			Seasonal phase and color change C4	0.21	0.05
			Space creation and scale coordination C5	0.09	0.02
			Harmony between plant community and surrounding environment C6	0.09	0.02
	Ecological value B2	0.62	Plant species diversity C7	0.41	0.26
			Life-type diversity C8	0.10	0.06
			Plant health status C9	0.15	0.09
			Environmental amelioration capacity C10	0.28	0.18
			Capability of resisting C11	0.06	0.04
	Social value B3	0.14	Native Plant Ornamental Characteristics C12	0.15	0.02
			Cultural Symbol Connotation C13	0.07	0.01
			Conservation and Utilization of Old and Valuable Trees C14	0.11	0.01
			Stayability C15	0.03	0.00
			Completeness of Supporting Facilities C16	0.23	0.03
			Safety C17	0.36	0.05
			Landscape Comfort C18	0.05	0.08

### Calculation of the comprehensive evaluation indices

In this study, 30 faculty members and students with backgrounds in horticulture and landscape architecture were invited to rate the qualitative indicators of 40 sample planted landscapes through a questionnaire. The participants comprised 15 current graduate and undergraduate students with at least an undergraduate degree, and 15 faculty members and industry practitioners with more than five years of relevant practical experience. All raters had received training in plant landscape design and evaluation, urban green space ecological planning, and related disciplines, ensuring the scientific rigor and representativeness of the evaluation. Participants were recruited through targeted invitations, with selection criteria including professional background, practical experience, and the ability to accurately assess plantscape characteristics.

To verify the reliability of the scoring data, an internal consistency test was conducted on the scoring data. Cronbach’s alpha coefficient of the qualitative index scores was 0.853, indicating that the questionnaire scores had high internal consistency and reliability. In addition, the stability and credibility of the data were enhanced by employing multiple independent raters and calculating the consistency index.

The calculation of the comprehensive evaluation index was based on the research findings of Shi Y. and Mao B. et al. [10,34] and incorporated the weights determined by the experts along with the sample scoring results to systematically evaluate the urban green space plantscape in Zhengzhou City. The formula for the comprehensive evaluation index is as follows:


$$B=\sum_{i=1}^{n}Ci\times Xi$$ (12)

$$CEI=\frac{B}{B0}\times 100\%$$ (13)

where B  represents the comprehensive evaluation index, $C_{i\;\;}$ represents the weight of the index, $X_{i\;\;}$ represents the score of sample i under the index, *n* represents the total number of evaluation indices, CEI represents the comprehensive evaluation index of the plant landscape of a sample, and $B_{\text{0}\;\;}$ represents the ideal composite score of a sample, which is the sum of the multiplication of the best scoring value of each factor and the corresponding total weight value [26].

On the basis of the calculation results, the overall evaluation of the plant landscape of urban green space in Zhengzhou City was carried out, and the quality of the plant landscape was categorized into four grades: Grade I (Excellent), Grade II (Good), Grade III (Average), and Grade IV (Poor) with reference to the classification standard of Crawford, K.M. et al. [24] (Table 8).

**Table 8:** Landscape quality scale.

CEI(%)	>80	80–60	60–45	<45
Landscape quality grade	I	II	III	IV

### SBE landscape evaluation method

In this study, the public aesthetic perceptions of plant landscape units in urban green spaces in Zhengzhou City were quantitatively evaluated via the landscape degree estimation (SBE) method. The SBE questionnaire survey was conducted online, and the sample sites and plant landscape photographs used in the evaluation were consistent with the AHP landscape evaluation system.

Photos of the plantscape were taken from May to October 2023 in Zhengzhou City. The photographed sites cover typical green space types, such as park green space, protective green space, subsidiary green space, square green space, and regional green space. To ensure the quality of the photographs and the consistency of the evaluations, a standardized framing scale (observation distance of 10–15 m) was employed during the shooting process. Photographs were taken under sunny weather and uniform lighting conditions to ensure that each image primarily represented a single plant landscape unit, minimizing the influence of environmental interferences on the visual experience. The SBE questionnaire consisted of 40 photographs of plant landscape units, all produced in a standardized format as a PowerPoint presentation (PPT). The introduction to the questionnaire provided a detailed explanation of the scoring criteria, and participants were asked to rate each photo based on their personal subjective perception using a 7-point scale, where 1 indicated “extremely unattractive” and 7 indicated “extremely attractive.” During the slide show, each photo was displayed for 10 s, with a 5-s transition between images, and the presentation was uniformly hosted by the researcher to maintain a consistent playback order and minimize external interference during the evaluation process. The participants were recruited through an online platform, and the screening criterion was adults with nongardening and landscape architecture professional backgrounds to ensure that the aesthetic evaluation better reflected the perceptions of the general public. A total of 30 participants were recruited, aged between 20–55 years, with a roughly balanced sex ratio, and randomly divided into 3 groups to complete the evaluation independently. The questionnaires were submitted online to ensure the immediacy and completeness of the data collection.

Referring to the research methods of Wang Y. [35], Gao Z. [36], Lichun Mo [5], and other scholars, this study standardized the collected rating data by converting the mean and standard deviation of the scoring results for each photograph. The standardized beauty value (SBE value) was then calculated to reflect the relative aesthetic level of each plant landscape unit within the context of public aesthetics. The calculation formula is as follows:


$$Z_{ij}=\frac{R_{ij}-{\bar{R}}_{j}}{S_{j}}$$ (14)

$$Z_{i}=\frac{\sum_{j=1}^{N_{i}}Z_{ij}}{N_{i}}$$ (15)

where ${\;Z}_{ij\;}$ represents the standardized value assigned by the j-th evaluator for the i-th landscape sample, ${\;R}_{ij\;}$ denotes the score given by the j-th evaluator for the i-th landscape sample, $\overset{―}{R_{j}}$ is the average score given by the j-th evaluator across all landscape samples, and ${\;S}_{i\;}$ refers to the standard deviation of the scores given by the j-th evaluator across all landscape samples. $Z_{i\;\;}$ is the standardized score of the i-th landscape sample, which corresponds to the SBE score, and $N_{i\;}$ represents the total number of evaluators assessing the i-th landscape sample. The higher the standardized score ($Z_{i\;}$>0) is, the better the landscape quality. Conversely, a lower standardized score (${\text{Z}}_{\text{i}\;}$≤0) indicates lower landscape quality [7,37,38].

To ensure the transparency and reproducibility of the study, the content of the questionnaire used in the SBE beauty evaluation is listed in the appendix at the end of the paper (Appendix 2 in S1 File). To test the consistency of results between the two evaluation methods (AHP composite evaluation and SBE beauty rating), Spearman’s rank correlation analysis was employed in this study. The analyses included: (1) testing the correlation between the AHP evaluation results and the SBE score results based on the composite scores of the 40 planted landscape samples [39], and (2) testing the consistency between the AHP and SBE methods in the ranking of green space types based on the composite scores of the five different green space categories. All correlation analyses were conducted using IBM SPSS Statistics 27.0 software, employing two-tailed tests with the significance level set at p < 0.05.

## Results

### Evaluation results of plant diversity in Zhengzhou city

According to the results of the plant diversity index, there were significant differences in the diversity indices of the five types of green space types in Zhengzhou City in the tree, shrub, and herbaceous layers (Table 9). In the tree layer, the park green space exhibited the best performance, with all four diversity indices being higher than the average value, indicating a significant advantage [40]. In contrast, park greenspaces were relatively rich in arbor species, whereas square sites and regional greenspaces presented lower arbor plant diversity indices overall. Specifically, ancillary green spaces ranked highest in terms of the Margalef species richness index, while park green spaces performed best in terms of the Simpson diversity index and Shannon-Wiener diversity index, and plaza lands presented a high Pielou evenness index but a low overall arborvitae diversity index. The diversity index results for the shrub layer were similar to those observed for the tree layer, with park green spaces continuing to lead significantly across all indices. Ancillary green spaces ranked second in the Margalef species richness index, Simpson diversity index, and Shannon-Wiener diversity index. In the herbaceous layer, the diversity indices of park green spaces and protective green spaces were both above the overall average, with park green spaces maintaining the highest rankings. In contrast, plaza sites ranked lowest across all indices, indicating a notable deficiency in herbaceous plant diversity. Overall, park green space has an overall advantage in terms of plant diversity, while square land and regional green space have a lower level of diversity, especially in the tree and herb layers, which needs to be improved.

**Table 9:** Index of the diversity of plants in different types of green spaces in Zhengzhou.

Plant categories	Type of green space	The MargalefIndex	The Simpson Index	The Shannon-Wiener index	Pielou index number
Trees	Park green spaces	8.26	0.97	3.62	0.88
	Protective green spaces	7.46	0.95	3.35	0.83
	Square green spaces	5.16	0.94	3.10	0.88
	Affiliated green spaces	8.63	0.95	3.54	0.79
	Regional green spaces	5.75	0.94	3.17	0.86
	Average value	7.05	0.95	3.36	0.85
Shrubs	Park green spaces	6.52	0.93	3.06	0.71
	Protective green spaces	4.25	0.89	2.59	0.67
	Square green spaces	4.99	0.88	2.56	0.65
	Affiliated green spaces	5.41	0.91	2.82	0.63
	Regional green spaces	3.53	0.90	2.67	0.74
	Average value	4.94	0.90	2.74	0.680
Herbaceous	Park green spaces	19.89	0.893	3.01	0.61
	Protective green spaces	18.88	0.91	3.20	0.63
	Square green spaces	13.01	0.76	2.32	0.50
	Affiliated green spaces	20.16	0.88	2.96	0.55
	Regional green spaces	13.28	0.94	3.18	0.68
	Average value	17.04	0.88	2.94	0.60

### Evaluation results of the AHP method

**Fig 2:**
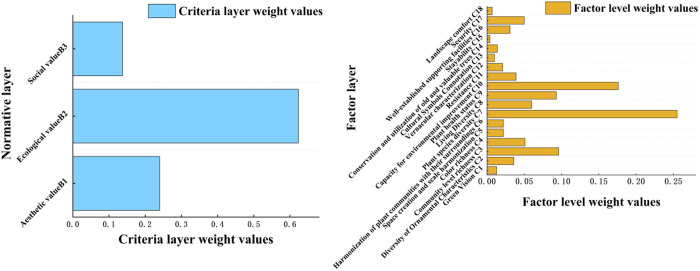
Ranking of total weights of guideline layer and factor layer for comprehensive evaluation of plant landscape in Zhengzhou city green space.

Using Yaahp v11.0 software, the weight values of each indicator within Zhengzhou’s urban green space plant landscape evaluation system were calculated. A comprehensive analysis of the weighting results revealed the following: at the criterion level, ecological value ranked highest, followed by aesthetic value and then social value. At the factor level, the top three indicators were plant species diversity, environmental improvement capacity, and community structure richness. According to the comprehensive evaluation model for Zhengzhou’s urban green spaces, the lowest-ranked indicators were primarily associated with social value, with landscape comfort and stayability occupying the final two positions (Fig 2).

On the basis of the weight values of each indicator and the actual measured values of Zhengzhou’s urban green spaces, the plant landscape quality evaluation results were calculated and summarized (Table 10). An analysis of the data revealed the following: Grade I plots accounted for 10% (4 plots), Grade II plots accounted for 57.5% (23 plots), and Grade III plots accounted for 32.5% (13 plots). Only 5 plots achieved an excellent quality level (CEI > 80%), whereas the remaining plots were rated as either good or average.

**Table 10:** AHP comprehensive evaluation results of the green space plant landscape in Zhengzhou.

Sample square number	Comprehensive score	CEI value	Landscape quality grade	Ranking
GY7	8.44	84.42%	I	1
GC1	8.31	83.13%	I	2
FS1	8.02	80.17%	I	3
GY2	8.01	80.12%	I	4
FS5	7.96	79.58%	II	5
QY6	7.54	75.39%	II	6
FH8	7.23	72.31%	II	7
FS6	7.21	72.08%	II	8
GY4	7.19	71.88%	II	9
GY5	7.17	71.68%	II	10
GC2	7.06	70.58%	II	11
QY4	7.01	70.05%	II	12
FH6	6.99	69.88%	II	13
FH5	6.90	68.98%	II	14
FH3	6.88	68.79%	II	15
FH4	6.72	67.18%	II	16
QY3	6.71	67.11%	II	17
QY7	6.67	66.65%	II	18
GY6	6.61	66.09%	II	19
GY1	6.56	65.59%	II	20
FH7	6.44	64.38%	II	21
FS3	6.40	63.99%	II	22
GY8	6.32	63.22%	II	23
QY5	6.20	62.02%	II	24
QY8	6.10	60.96%	II	25
GC8	6.08	60.76%	II	26
FS8	6.02	60.19%	II	27
GC7	5.98	59.80%	III	28
FS4	5.87	58.68%	III	29
GY3	5.70	57.03%	III	30
FH1	5.68	56.79%	III	31
QY2	5.60	56.02%	III	32
FS2	5.50	55.00%	III	33
QY1	5.45	54.52%	III	34
GC4	5.40	53.96%	III	35
GC3	5.27	52.73%	III	36
GC5	5.19	51.92%	III	37
FH2	5.14	51.39%	III	38
FS7	5.07	50.67%	III	39
GC6	4.72	47.22%	III	40

**Fig 3:**
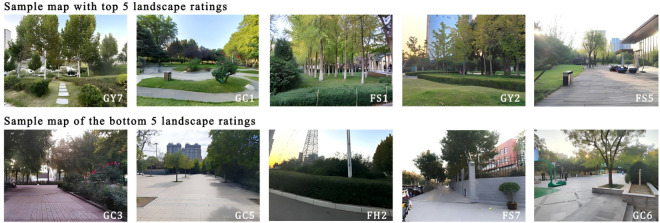
Comparison of the current status of the top 5 and bottom 5 samples. All research photographs referenced in this article were taken by the author during field investigations, ensuring their originality and legitimacy. No third-party copyright issues exist, thus no additional copyright statements are required.

Fig 3 presents a photo comparison of the top 5 and bottom 5 plots evaluated via the AHP method. The photographs of the five lowest-ranked plots demonstrate problems such as low plant diversity and repetitive landscape design within the urban green spaces of Zhengzhou. These observations emphasize the necessity for enhanced planning and refinement of the city’s plant landscape structures.

In the current research, plots were grouped according to the type of green space, with the average values calculated from eight sample plots for each category. The outcomes, summarized in Table 11, indicate that park green spaces achieved the highest evaluation scores, showing a clear advantage over the other four green space categories. This suggests that, under similar physical conditions, plant landscapes with higher biodiversity tend to achieve superior evaluation outcomes.

**Table 11:** The average value of the comprehensive evaluation of the plant landscapes of different urban green space types in Zhengzhou via the AHP method.

Classification of urban green space types	Composite score mean	CEI mean	Ranking
Park green spaces	7.00	70.00%	1
Affiliated green spaces	6.50	65.05%	2
Protective green spaces	6.50	64.96%	3
Regional green spaces	6.41	64.09%	4
Square green spaces	6.00	60.01%	5

### Evaluation results of the SBE method

By standardizing the data from the online questionnaire, the SBE values for 40 plant landscape plots were calculated. The detailed evaluation results are presented in Table 12. Analysis of the data reveals that 17 plots (42.5% of all evaluated plots) have SBE values greater than 0. Among these, 5 are park green spaces, 4 are ancillary green spaces, 4 are protective green spaces, 3 are plaza green spaces, and 1 is a regional green space. Conversely, 23 plots (57.5% of all evaluated plots) have SBE values less than 0. These spaces include 3 park green spaces, 4 affiliated green spaces, 4 protective green spaces, 5 square green spaces, and 7 regional green spaces.

**Table 12:** SBE comprehensive evaluation results of the green space plant landscape in Zhengzhou.

Sample number	SBE value	Ranking	Sample number	SBE value	Ranking
GY7	1.54	1	GC5	−0.19	21
GC1	1.13	2	GY3	−0.23	22
GY2	0.97	3	GC3	−0.25	23
FS1	0.96	4	QY3	−0.26	24
FS5	0.87	5	FS4	−0.27	25
FH3	0.85	6	QY7	−0.27	26
FS8	0.84	7	FS2	−0.33	27
GC2	0.83	8	QY5	−0.37	28
FH8	0.83	9	GY1	−0.39	29
FH6	0.77	10	FS7	−0.52	30
GY5	0.62	11	QY1	−0.61	31
GY6	0.51	12	GC8	−0.64	32
FH5	0.39	13	FH7	−0.67	33
GY4	0.35	14	QY8	−0.72	34
QY4	0.30	15	QY2	−0.72	35
GC7	0.22	16	QY6	−0.77	36
FS6	0.01	17	GC4	−0.88	37
FH4	−0.05	18	FH2	−0.96	38
FS3	−0.07	19	GY8	−1.04	39
FH1	−0.16	20	GC6	−1.64	40

On the basis of the SBE evaluation results, this study conducted a classification analysis by urban green space type, as shown in Table 13. The results indicate that park green spaces achieved the highest scenic beauty scores, significantly outperforming the other four types of green spaces. In contrast, regional green spaces ranked the lowest in scenic beauty evaluation.

**Table 13:** The average value of the comprehensive evaluation of the plant landscapes of different urban green space types in Zhengzhou via the SBE method.

Urban green space type	SBE value	Ranking
Park green spaces	0.29	1
Affiliated green spaces	0.19	2
Protective green spaces	0.13	3
Square green spaces	−0.18	4
Regional green spaces	−0.43	5

### Comparison of the evaluation results between the AHP method and SBE method

By comparing the evaluation results of the AHP and SBE methods (Tables 10 and 12), both consistencies and differences in the rankings of the plant landscape plots were identified. Among the top 5 plots, 3 plots (GY7, GC1, FS5) ranked identically in both methods, with a consistency rate of 60%. Among the bottom 5 plots, 2 plots (FH2, GC6) had identical rankings, yielding a consistency rate of 40%. Further analysis revealed that, according to the AHP method, the top 5 plots included park green spaces, affiliated green spaces, and square green spaces. The bottom five plots consisted of protective green spaces, ancillary green spaces, and plaza green spaces. In contrast, the SBE method’s evaluation showed that the top five plots included park green spaces, ancillary green spaces, protective green spaces, and plaza green spaces, while the bottom five consisted of ancillary green spaces, regional green spaces, protective green spaces, and plaza green spaces. A comparative analysis indicates that, among the top five plots, both methods included park green spaces, ancillary green spaces, and plaza green spaces, but none included regional green spaces. Among the bottom five plots, both methods consistently identified plaza green spaces, protective green spaces, and ancillary green spaces, with no park green spaces appearing.

At the green space type level (Tables 11 and 13), the rankings produced by the AHP and SBE methods are fully consistent for the top three types: park green spaces, ancillary green spaces, and protective green spaces. However, the rankings of the last two types differ, with the order of regional green spaces and plaza green spaces reversed. Overall, the AHP and SBE methods exhibit high consistency in the general evaluation direction, though differences in specific rankings are evident, reflecting methodological distinctions and subjective preferences. This indicates that each method has strengths: the AHP method emphasizes the comprehensiveness of objective indicators, whereas the SBE method relies more on evaluators’ subjective perceptions.

Spearman correlation analysis revealed a significant positive correlation between the AHP comprehensive evaluation scores and the SBE beauty scores (correlation coefficient rs = 0.738, p < 0.001). Additionally, a strong positive correlation was observed between the AHP and SBE methods in the ranking of different urban green space types (correlation coefficient *r*_*s*_ = 0.900, *p* = 0.037), reaching statistical significance at the 0.05 level. These results indicate a high degree of consistency between the two methods in evaluating the quality of planted landscapes and in the comprehensive evaluation of different green space types (Table 14).

**Table 14:** Spearman rank correlation analysis between AHP and SBE evaluation scores at the sample level and the green space type level.

Comparison level	Variables	AHP result	SBE result
	AHP result	1	0.738**
Spearman rank correlation analysis of AHP – SBE evaluation results (sample level)	SBE result	0.738**	1
	Number of samples	40	40
	AHP result	1	0.900*
Spearman’s hierarchical correlation analysis of AHP – SBE evaluation results (green space type level)	SBE result	0.900*	1
	Number of green spacetypes	5	5

**Note:** *p < 0.05, **p < 0.01, two-tailed.

In order to further explore the differences between different green space types in terms of the comprehensive plant landscape score (AHP evaluation) and the perceived beauty score (SBE evaluation), this study used the Kruskal-Wallis H-test to conduct non-parametric inferential analysis [34]. The comprehensive AHP score test results indicated that there were no statistically significant differences among the various green space types (*H* = 4.579, *p* = 0.333). Similarly, the SBE aesthetic score test results suggested that differences in public subjective perception across green space types were also not significant (*H* = 6.184, *p* = 0.186), suggesting that the overall variation in public aesthetic assessment of plant landscapes among different green space categories was relatively minor (Table 15). Collectively, these findings suggest that the urban green space plant landscapes in Zhengzhou demonstrate a high degree of uniformity and standardization across different types, as reflected in both expert evaluations and public aesthetic judgments.

**Table 15:** Results of the Kruskal-Wallis H test for the landscape scores of different green space types.

Evaluation methodology	H-value	P value	significance level
AHP composite score	4.579	0.333	insignificant(*p* > 0.05)
SBE Beauty Score	6.184	0.186	insignificant(*p* > 0.05)

## Discussion

### Structural characteristics and type differences in plant diversity in the urban green spaces of Zhengzhou city

A total of 596 species of vascular plants belonging to 110 families and 357 genera were recorded in the urban green space of Zhengzhou City in this field survey, reflecting the high level of plant species diversity in this area. The results were essentially consistent with those of Qi R [41], indicating that this sample survey has a strong scientific basis and comparability in terms of spatial distribution and representativeness. On the whole, the plant species of urban green space in Zhengzhou City are relatively rich, which can better support the multifunctional demand of urban ecosystems.

There are significant differences in plant diversity indicators among different types of green space. Park greenspace has the highest species diversity at the three levels of trees, shrubs, and herbs. It is the type of green space with the richest plant configuration and the most complete landscape structure. Subsidiary green space ranked second in diversity, particularly in the tree and shrub layers, especially in areas surrounding research nurseries, institutional yards, and transportation facilities, where plant introductions and configurations were more diverse [42]. Protective green space demonstrated clear advantages in the herbaceous layer and is commonly applied in high-voltage corridors, slopes, and other specialized functional areas, where ground cover plants are extensively planted to achieve both ecological restoration and landscape beautification. In contrast, regional green spaces and plazas exhibited relatively low plant diversity, with plazas performing particularly poorly due to the large proportion of hard surfaces, limited green areas, and insufficient herbaceous plant allocation, resulting in weaker ecological and landscape functions [43]. Similar patterns have been observed in international research. Investigations conducted in cities such as Berlin, Germany, and Stockholm, Sweden, have found that green spaces designated for research, conservation, or public service purposes tend to exhibit higher levels of plant richness and evenness [44,45]. These findings indicate that the relationship between green space types and plant diversity patterns displays certain global consistencies.

From the perspective of plant life-types, significant differences were observed in the diversity characteristics across different vegetation layers. In the tree layer, the Margalef species richness index was highest in subsidiary green spaces, a finding consistent with the studies of Vakhlamova and Su et al. [46,47], and likely associated with the diversified species introductions and long-term management practices characteristic of nursery-type subsidiary green spaces. The shrub layer also exhibited the greatest diversity in ancillary green spaces, where service facilities and traffic nodes are often complemented by colorful and varied ornamental shrubs to enhance visual appeal [48]. In the herbaceous layer, the diversity index was significantly higher in protective green spaces compared to other types, with ground cover plants such as maitake and daylily being widely employed to promote both ecological stability and landscape aesthetics [49]. On the other hand, the diversity index of square green space in the herbaceous layer was the lowest, mainly because of the limited area of green space and the neglect of ground cover configuration [50].

In summary, different types of green spaces in Zhengzhou City presented systematic differences in terms of plant life type structure and diversity distribution, reflecting the joint influence of green space functional attributes, spatial patterns, and management styles on plant landscape patterns. This study provides important data support and a practical basis for urban green space classification construction, differentiated plant configurations, and ecological function optimization.

### Analysis of the AHP-SBE comprehensive evaluation results of the plant landscape

In this study, the AHP method and SBE method were used to evaluate 40 typical planted landscapes in Zhengzhou City, and the AHP method was used to construct an evaluation system based on the three criteria of ecology, aesthetics, and society. The results revealed that the ecological value had the highest weighting, which emphasized the key role of urban green space in maintaining ecological balance and environmental regulation. This result is not only consistent with the viewpoints of Wu J. and Wang L. et al. [38,51] but also highlights the fundamental role of urban green spaces in addressing challenges such as the heat island effect, air pollution, and habitat fragmentation under current urban environmental pressures. The aesthetic value ranked second, underscoring the significance of plantscapes in enhancing the urban visual environment and improving residents’ living experiences, consistent with the findings of Teimouri R. and Cui et al. [52,53]. The relatively low weighting of social value may be attributed to its more implicit nature, making it difficult to quantify directly. Additionally, limited public awareness often results in its manifestation through indirect contributions to cultural identity, historical significance, and community cohesion [54]. International studies have also shown that urban green spaces play multiple roles in providing ecological services, aesthetic experiences, and social well-being, and Tzoulas K et al. (2007) noted that green infrastructure not only affects ecosystem services but also positively affects the health and social well-being of residents [55]. Kabisch N. et al., in a multicountry study in Europe, further confirmed that the synergistic development of ecological, aesthetic, and social functions is the key to green urbanization [56]. Synergistic development is an important direction of green city construction. Therefore, the construction of green spaces in Zhengzhou City should align with the international green city concept, and while safeguarding ecological functions, it should continue to promote the integrated enhancement of landscape aesthetics and social functions.

In the factor-level evaluation, plant species diversity holds the highest weight, serving as a core indicator whose richness directly influences both the ecological stability and aesthetic quality of green spaces [57,58]. Its primary weighting is attributed to its dual role in supporting ecological functions and enhancing aesthetic appeal, forming the foundation for green spaces to deliver ecological services and visual attractiveness [7,59]. The ability to improve the environment ranks second, reflecting the important role of urban green space in ecological services such as air purification and microclimate regulation [2]. Especially in the context of rapid urbanization, the public and planners continue to pay more attention to the environmental regulation benefits of green space, which increases the weight of this indicator in comprehensive evaluations. Community-level richness ranked third, mainly reflecting the contribution of multi-level plant configuration in enhancing the three-dimensional and visual variability of the landscape. Although its ecological function is somewhat inferior to that of species diversity and environmental improvement capacity, in landscape design practice, it holds significant value in shaping the spatial perception of the landscape and enhancing its ornamental qualities, thereby warranting a certain evaluation weight. In contrast, the weight assigned to indicators related to landscape comfort, stayability, and other aspects of human experience is relatively low, which may be attributed to their susceptibility to subjective factors and the potential for individual bias during the evaluation process. Moreover, the impact of these indicators generally relies on the supporting facilities and the spatial use of the green space. Their direct relationship with the plant configuration is relatively weak. Therefore, their significance in structural evaluation remains limited. Overall, the findings of this study suggest that, in efforts to optimize plant landscapes in urban green space development, priority should be placed on enhancing plant species diversity, ecological regulatory functions, and community structure complexity. This approach aims to achieve the integrated development of ecological performance, visual appeal, and spatial adaptability, thereby supporting the high-quality advancement of urban green space systems.

The SBE method reflects the aesthetic experience of urban residents in plant landscapes from the perspective of the subjective perceptions of the public. The analysis of the SBE scores of the 40 sample squares revealed that 57.5% of the sample squares had scores lower than 0, indicating that there is still much room for improvement in the overall aesthetic experience of plant landscapes in urban green spaces in Zhengzhou City. Optimization suggestions include increasing plant species, enriching plant structure forms, and strengthening color combinations and seasonal changes to increase the ornamental value and diversity of the landscape [60]. Among them, the SBE score of regional green space is the lowest, mainly because its naturalized management style results in a messy plant structure and a lack of systematic maintenance [61,62]. Overseas studies have demonstrated that moderately managed naturalized green spaces—such as the concept of “managed nature” in North America and Northern Europe—can significantly enhance the public’s visual experience and landscape identity through measures such as the introduction of native ornamental plants and the implementation of landscape guiding facilities [63,64].

In summary, the AHP method prioritizes a structured and logical approach, making it appropriate for comprehensive evaluation involving multiple indicators. In contrast, the SBE method highlights perceptual experience and subjective aesthetic judgment, offering quick insight into public impressions of plant landscapes. Each method has distinct strengths and serves as a complement to the other, together offering a well-rounded and varied perspective for assessing urban landscape quality. This combination also delivers valuable scientific guidance and practical reference for improving green space systems.

### Comparative analysis of the AHP and SBE methods in plant landscape evaluation

In this study, 40 typical plant landscape samples from Zhengzhou City were comprehensively evaluated via the AHP and SBE methods, and the results revealed that the evaluation results of the two methods were somewhat consistent. In the ranking of single landscape samples, samples with higher AHP scores also mostly received higher beauty scores in SBE, indicating that the two methods have relatively consistent trends in identifying overall landscape quality. However, due to significant differences in the evaluation dimensions and focal points of the two methods, certain inconsistencies were observed: the AHP method emphasizes the systematic analysis of ecological attributes, encompassing dimensions such as plant diversity, community structure, and environmental improvement [59,65], whereas the SBE method focuses on the evaluator’s subjective aesthetic perception, which is more influenced by factors such as photographic angle, seasonal condition, and visual impression.

To further quantify the evaluation consistency between the two methods, this study employed Spearman rank correlation analysis to test the relationship between the AHP composite score and the SBE score. The results indicated a moderate positive correlation between the two, suggesting that despite differences in evaluation logic, a certain degree of consistency existed in the overall ranking trend. This finding provides empirical support for the complementary application of the two methods in the evaluation of plant landscapes.

At the level of green space types, the rankings of AHP and SBE for the five types of green spaces were consistent in the top 3 (park green space > subsidiary green space > protective green space), but there was a difference in the rankings of regional green space and square land [66]. Regional green space scored low in the SBE method because of disorganized plant community structure and insufficient maintenance and management [67,68] but ranked relatively high in the AHP method because of its species richness and strong ecological function [32,69]. Plaza land, on the other hand, has a weak ecological function due to a high proportion of hard paving and insufficient plant diversity and is ranked low in the AHP method [70] but relatively high in SBE because of its neat and orderly layout and good public facilities, which enhance the visual experience [39]. This result is consistent with Wang L.’s findings on the perceived variability of multiple functions of green space [38]. In addition, although some sample squares were rich in plant species, the overall ornamental experience was poor due to chaotic plant configurations and a lack of structural hierarchy, resulting in low SBE scores. This finding indicates that although species richness is the basis of evaluation, the role of spatial organization and landscape design quality in landscape perception is equally critical.

The Kruskal-Wallis H test was further used to analyze the difference between the AHP and SBE scores of different green space types, and the results revealed that the two types of scores did not reach statistical significance (p > 0.05) among the green space types. This reflects that different types of green spaces in Zhengzhou tend to be balanced in terms of landscape quality as a whole. This phenomenon may stem from urban green space construction tending toward standardization in recent years, but it also suggests that the differentiated characteristics between green space types have weakened and that there is a certain degree of landscape homogenization. Therefore, in the future, green space planning should pay more attention to functional orientation and implement diversified plant allocation strategies for different green space types to increase the ecological resilience and landscape diversity of the green space system.

In terms of methodological characteristics, the AHP method emphasizes scientific logic and an indicator weighting structure, making it suitable for multifactorial rational analysis and the formulation of policy proposals [71]. In contrast, the SBE method is characterized by its operational simplicity, facilitating the rapid collection of public aesthetic evaluations and feedback on social acceptance, and is thus well-suited for the perceptual verification of landscape optimization strategies [72]. The joint use of the two can realize the complementarity of rational analysis and perceptual experience, which can help improve the comprehensiveness and scientific validity of plant landscape evaluation.

The integrated application of evaluation methods is also widely employed in international urban green space research. For example, in the management of urban green spaces in the United States and Australia, quantitative ecological analyses are often combined with public perception assessments to achieve a balance between scientific rigor and practical application [73,74]. This study not only advances the comprehensive evaluation methodology of urban plant landscapes through the parallel application of AHP and SBE but also provides empirical support for the differentiated construction and landscape optimization of urban green spaces in central China.

## Limitations and outlook

This study integrates the AHP and SBE methods, synthesizing subjective and objective evaluation approaches to enhance the scientific rigor and practical guidance value of plant landscape quality assessment [7]. However, certain limitations remain within the research process. First, in the AHP method, the selection of indicators and the assignment of weights are inevitably influenced by the subjective judgment of experts. Although efforts were made to control for this through expert consultations and questionnaire surveys, the standardization and objectivity of the process still require further improvement. Second, the SBE method is based on the subjective perceptions of evaluators, making it vulnerable to influence from individual aesthetic preferences, cultural background, and varying levels of knowledge, which may introduce bias into the evaluation outcomes [10,18,70]. Furthermore, factors such as a limited sample size, a one-time survey centered on specific seasons, and the uneven spatial distribution of urban green spaces may restrict the generalizability and applicability of the study’s findings.

Future research should be further strengthened in the following areas: first, by expanding the survey scale to include a wider range of cities and green space types, thereby enhancing the representativeness of the samples; second, by extending the survey period and conducting multitemporal dynamic monitoring across different seasons and years, to more accurately capture the seasonal dynamics of plant landscape changes; third, by improving the standardization of data processing through the integration of multi-source data and the adoption of a quantitative index system, thus enhancing the scientific rigor and comparability of the evaluations; and fourth, by deepening international research collaboration and development efforts. Fourth, international comparative research should explore the similarities, differences, and applicability of plant landscape evaluation systems in different countries and regions to promote the organic integration of local and international standards. Moreover, the AHP method and SBE method show some complementarity in the comprehensive evaluation of the landscape of plants, with the former emphasizing ecological functionality and systematicity and the latter highlighting public aesthetic perception [75]. Future research can explore the establishment of a comprehensive evaluation framework that integrates both quantitative indicators and subjective perceptions. By incorporating weight optimization, perceptual feedback mechanisms, and dynamic monitoring approaches, the scientific rigor and practical applicability of plant landscape quality assessments can be further enhanced, thereby providing more systematic and accurate support for the development of green cities.

## Conclusion

On the basis of the AHP method and SBE method, this study constructed a comprehensive evaluation system of the plant landscape in urban green spaces in Zhengzhou City and systematically measured and graded 40 plant landscape samples. The results revealed that the diversity indices of park green space in the tree layer, shrub layer, and herb layer were all greater than the average level, indicating obvious advantages and reflecting the important role of long-term development and functional orientation in the accumulation of plant diversity. The comprehensive evaluation system, constructed based on the three criteria of ecological value, aesthetic value, and social value, effectively captures the quality characteristics of urban green spaces. Among these, the level of plant diversity exhibits a strong correlation with the comprehensive score, thereby confirming the critical role of plant diversity in enhancing the quality of green space landscapes.

The AHP method and the SBE method were highly consistent in terms of the rankings of the plant landscape samples and the rankings of the green space types in general, but there were some differences in the specific details. Spearman rank correlation analysis revealed that there was a moderate positive correlation between the AHP composite score and the SBE beauty score, which verified the complementary nature of the two methods in the evaluation of landscape quality. Moreover, the results of the Kruskal-Wallis H test revealed that different green space types did not result in statistically significant differences in composite scores or beauty scores, indicating that urban green spaces in Zhengzhou tend to be balanced in terms of the comprehensive quality of plant landscapes and public perceptions of aesthetics; however, this finding also suggests that there is a certain degree of homogenization of the green space system in terms of plant abundance, landscape hierarchy, and functional characteristics. Further analysis revealed that some plots with moderate plant diversity exhibited lower overall rankings, which may be attributed to poor spatial design, insufficient visual coherence, or lower levels of maintenance and management. This phenomenon underscores that while plant diversity serves as a critical foundation for urban green space development, reasonable spatial organization, a clear hierarchical structure, and a high-quality landscape experience are equally important factors influencing the quality of planted landscapes.

In the planning and construction of urban green spaces in the future, more attention should be given to the synergistic optimization of plant diversity protection and landscape space design. Species richness should be improved, community-level optimization should be performed, native plants should be protected, and the control of exotic species as the core should be implemented to build a green infrastructure network with strong connectivity and composite functions; at the same time, attention should be given to the simultaneous enhancement of the aesthetics of the landscape space and ecological functions to avoid the phenomenon of monotonization of the green space and homogenization of functions. Drawing on the experience of international green city construction, dynamic monitoring, and scientific assessment mechanisms should be established, quantitative index analysis should be combined with public perception evaluation, and the continuous improvement of green space systems in the dual dimensions of ecological resilience and landscape experience should be promoted. Through the synergistic optimization of ecological, aesthetic, and social functions, Zhengzhou City is expected to develop a high-quality urban ecological space that embodies local characteristics while achieving international competitiveness within the global green city development trend. The findings of this study not only offer a theoretical reference and practical foundation for the optimization of urban green space plant landscapes in Zhengzhou but also provide valuable insights for enhancing plant diversity and improving urban green space landscapes in other cities undergoing green transformation within the central region and across the country, thereby demonstrating significant application value and broader relevance.

## Supporting information

S1 File S1 Table. (DOCX). S2 File. (XLSX). S3 All the ethical parameters for human involvement in this study (PDF).
(ZIP)
