# Supplementary material for: Evaluation of urban green space plant landscape quality in Zhengzhou city using the AHP-SBE method
Source: PLoS One. 2025 Sep 26;20(9):e0329119. doi: 10.1371/journal.pone.0329119 (PMC12469112; doi:10.1371/journal.pone.0329119)

**Appendice 1**

**Table 1.** **Evaluation indicators and specific criteria for the plant landscape in Zhengzhou's urban green spaces.**

| **Target layer** | **Standardized layer** | **Factor level** | **Evaluation levels and scores** | | | | |
| --- | --- | --- | --- | --- | --- | --- | --- |
|  |  |  | **Excellent（8–10）** | **Good**  **（6–8）** | **Average**  **（4–6）** | **Poor**  **（2–4）** | **Very poor**  **（0–2）** |
| Comprehensive Evaluation of Zhengzhou Urban Greenland Plantscape A | Aesthetic value B1 | Green visibilityC1 | ≥60％ | 45％-60％ | 30％-45％ | 15％-30％ | ≤15％ |
|  |  | Diversity of ornamental characteristics C2 | Rich ornamental characteristics, with flowers, foliage, fruit, shape plants | With flowers, foliage, fruit and shape of plants in three of these categories | Two of the categories of plants with flowers, foliage, fruits, and shapes | Only one of the categories of flowering, foliage, fruiting, and formative plants | No flower, foliage, fruit, shape plants |
|  |  | Hierarchical richness of communities C3 | Very rich levels, ≥ 30 species of trees, shrubs and grasses, distinctive features | More abundant layers, with 21-29 species of trees, shrubs and grasses, and more distinctive features | General level, 11-20 species of trees, shrubs and grasses, general characteristics | Poor hierarchy, with 6-10 species of trees, shrubs and grasses, with no obvious characteristics | No hierarchy, ≤5 species of trees, shrubs and grasses, no special features at all |
|  |  | Seasonal phase and color change C4 | The landscape performance of each season is distinct, with strong visual appeal, rich color and natural transition | At least three seasons have unique landscape features, with good color matching and certain transition levels | Outstanding landscape performance only in one or two seasons, with reasonable color matching and bland visual effect of seasonal change | Most of the time the landscape performance is bland, lack of seasonal characteristics, single color, lack of change and sense of hierarchy, low visual appeal | Weak or the same landscape characteristics in each season, monotonous color, no transition level, poor sensory experience |
|  |  | Space creation and scale coordination C5 | Plant planting is very regular, space creation is very rich, with different types of space, and the scale is very coordinated | Spatial creation is reasonable, with one or two different spatial types, and the overall scale is comfortable | Spatial creation is not outstanding, only open or closed space, but the scale is coordinated as a whole | Overall bland, no obvious spatial characteristics, no obvious sense of scale | Chaotic planting, spatial disorganization, and very incongruous scale |
|  |  | Harmony between plant community and surrounding environmentC6 | The plant landscape is perfectly integrated with the surrounding environment, and the landscape is very harmonious | The plant landscape is similar to the surrounding environment and can reflect each other | The plant landscape is basically in harmony with the surrounding environment, and there is partial connection | The plant landscape has no obvious connection with the surrounding environment, but it is not abrupt | The plant landscape is isolated, without any connection with the outside world and very abrupt |
|  | Ecological value B2 | Plant species diversityC7 | Plant species ≥ 15 | 10-14 plant species | 6-10 plant species | 3-5 plant species | Plant species ≤ 2 |
|  |  | Life-type diversity C8 | With 8 different plant life types | 6 out of 8 different plant life types | Has 4 of the 8 different plant life types | Having 2 of the 8 different plant life types | Not having any plant or any plant life type |
|  |  | Plant health status C9 | Vigorous growth, full plant shape, no pests and diseases, glossy and bright leaves, normal development of flowers and fruits | Overall growth is good, with occasional diseases, a little wilting, and normal flowering and fruiting | Normal but slightly sparse growth, mildly infested with pests and diseases, slightly dull, partially wilted foliage | Poor growth, noticeably sparse or wilted, more pronounced pests and diseases, generalized wilting of foliage, widespread loss of foliage, and impeded flowering and fruiting | Plants in serious decline or near death, with total wilting of foliage, no normal flowering and fruiting, and loss of plant vigor |
|  |  | Environmental amelioration capacity C10 | Good improvement effect on the environment, and strong improvement ability, can achieve sustainable development | The plants in the community as a whole have the ability to conserve soil and water and absorb harmful gases | Only a single or individual plant has the ability to improve the environment | Does not show outstanding environmental amelioration, but does not cause pollution | The plant community does not have any ability to improve the environment and is prone to pollution |
|  |  | Capability of resisting C11 | Strong adaptability to various adverse environmental conditions, fast self-repair and excellent tolerance | Able to adapt to most of the environmental stresses, able to repair themselves quickly, and with good tolerance | Tolerant of adverse environmental conditions, slow to recover, show moderate tolerance | Showing low tolerance to adverse environmental conditions, poor resistance to pests and diseases, insufficient tolerance | Little or no ability to adapt to adverse environmental conditions, difficult to recover from adversity, very low tolerance and adaptability |
|  | Social value B3 | Native Plant Ornamental Characteristics C12 | The proportion of native plants is ≥80%, with strong visual attraction, significant landscape highlights in all seasons, and can be used as the core plants or landscape focal points | The proportion of native plants is between 60% and 80%, with a certain degree of ornamental, more obvious seasonal changes, and forming a good match with other plants | The proportion of native plants ranges from 40% to 60%, with medium ornamental value, insignificant seasonal changes, and need to be paired with other plants to emphasize their beauty | The proportion of native plants ranges from 20% to 40%, with low ornamental value, weak seasonal changes, and easy to be covered by other plants | Proportion of native plants ≤ 20%, poor visual performance, lack of seasonal changes, no sense of presence in the green space |
|  |  | Cultural Symbol Connotation C13 | The plants have deep historical and cultural deposits, are highly compatible with local culture, and the cultural connotation is widely disseminated | Plants have a certain cultural background or symbolic significance, a good fit with the local culture, and a certain degree of visibility of cultural connotations | The cultural significance of the plants is relatively common, generally associated with local culture, and the cultural connotation is less influential | The plants basically do not have significant cultural background or symbolic meaning, and have low relevance to the local culture and very low cultural influence | The plants do not have any cultural background or symbolic significance, do not fit in with the local culture, and have no cultural influence at all |
|  |  | Conservation and Utilization of Old and Valuable Trees C14 | There are many old and famous trees in the community, which are well protected and fully utilized, giving full play to their value | There are some old and famous trees in the community, and the existing trees are well protected | There are a small number of old and valuable trees in the community, but they are not completely protected or only individual valuable trees are protected and utilized | There are no old and famous trees in the community, and the existing trees have suffered some damage | There are no old and valuable trees in the community, and the existing trees have suffered serious damage |
|  |  | Stayability C15 | The landscape features are obvious, with space and facilities for viewing, strong ability to attract tourists to stop, and the tourists' stay time is ≥8min | Landscape with characteristics, with the conditions for tourists to stay for a short period of time, and the tourists' stay time is 6-7min | The landscape has certain characteristics, but there is not enough space or facilities for visitors to stay for 4-5min | The landscape is relatively boring, only a few tourists are willing to stop and watch, and the tourists' stay time is 2-3min | The landscape is monotonous and does not have space and facilities to stay and watch, not attracting tourists, and the tourists' stay time is ≤1min |
|  |  | Completeness of Supporting Facilities C16 | Complete variety, sufficient quantity, strong functionality, reasonable layout and good maintenance | A complete range of species, sufficient quantity, strong functionality, reasonable layout and good maintenance | General species, general quantity, general functionality, general layout, general maintenance | Lack of species, insufficient number, poor functionality, irrational layout and poor maintenance | Scarcity of species, very few in number, poor functionality, chaotic layout, poor maintenance |
|  |  | Safety C17 | Stable community structure, non-toxic, no thorns, no plant tilt, no invasive plants | Stable community structure, individual plants with poisonous thorns, no obvious inclination, fewer exotic plants | General community structure, some plants are poisonous and have thorns, some trees are leaning, a few exotic plants | Unstable community structure, obviously poisonous with thorns, a large number of dead branches and leaning, a high proportion of invasive plants | The community structure is seriously unstable, a large number of plants have thorns and poisonous, there are a lot of dead branches and broken trees, and a large number of invasive plants |
|  |  | Landscape Comfort C18 | Rich plant species, open space, appropriate lighting, strong ornamental properties, and long residence time | More plant species, more open space, more suitable light, better visual attraction | Average plant species and levels, fair use of space, poor lighting, not attractive enough | Single plant species, poor spatial design, sun exposure, short residence time | Lack of plant species, chaotic spatial design, too much light, unwilling to stay |

**Table 3. Mean value matrix and weight of criterion layer for comprehensive evaluation of urban green space plant landscape in Zhengzhou.**

| **Matrix 1** | **Aesthetic value** | **Ecological value** | **Social value** | **Weighted value** |
| --- | --- | --- | --- | --- |
| Aesthetic value | 1.00 | 0.33 | 2.00 | 0.24 |
| Ecological value | 3.00 | 1.00 | 4.00 | 0.62 |
| Social value | 0.50 | 0.25 | 1.00 | 0.14 |

In this matrix, *λmax* = 3.018, *C.I.* = 0.009, *R.I.* = 0.520, *C.R.* = 0.018 < 0.1, and the consistency test passes.

**Table 4. ‘Aesthetic value’ factor layer mean value matrix and weight.**

| **Matrix 2** | **Green visibility** | **Diversity of Ornamental Characteristics** | **Hierarchical richness of communities** | **Seasonal phase and color change** | **Space creation and scale coordination** | **Harmony between plant community and surrounding environment** | **weighted value** |
| --- | --- | --- | --- | --- | --- | --- | --- |
| Green visibility | 1.00 | 0.33 | 0.20 | 0.25 | 0.50 | 0.50 | 0.05 |
| Diversity of ornamental characteristics | 3.00 | 1.00 | 0.33 | 0.50 | 2.00 | 2.00 | 0.15 |
| Hierarchical richness of communities | 5.00 | 3.00 | 1.00 | 2.00 | 5.00 | 5.00 | 0.40 |
| Seasonal phase and color change | 4.00 | 2.00 | 0.50 | 1.00 | 2.00 | 2.00 | 0.21 |
| Space creation and scale coordination | 2.00 | 0.50 | 0.20 | 0.50 | 1.00 | 1.00 | 0.09 |
| Harmony between plant community and surrounding environment | 2.00 | 0.50 | 0.20 | 0.50 | 1.00 | 1.00 | 0.09 |

In this matrix, *λmax*=6.086, *C.I.*=0.017, *R.I.*=1.260, *C.R.*=0.014 <0.1, and the consistency test was passed.

**Table 5.‘Ecological value’ factor layer mean value matrix and weight.**

| **Matrix 3** | **Plant species diversity** | **Life-type diversity** | **Plant healthstatus** | **Environmental amelioration capacity** | **Capability of resisting** | **weighted value** |
| --- | --- | --- | --- | --- | --- | --- |
| Plant species diversity | 1.00 | 4.00 | 3.00 | 2.00 | 5.00 | 0.41 |
| Life-type diversity | 0.25 | 1.00 | 0.50 | 0.33 | 2.00 | 0.10 |
| Plant health status | 0.33 | 2.00 | 1.00 | 0.33 | 3.00 | 0.15 |
| Environmental amelioration capacity | 0.50 | 3.00 | 3.00 | 1.00 | 4.00 | 0.28 |
| Capability of resisting | 0.20 | 0.50 | 0.33 | 0.25 | 1.00 | 0.06 |

In this matrix, *λmax*=5.121, *C.I.*=0.030, *R.I.*=1.120, *C.R.*=0.027 was <0.1, and the consistency test was passed.

**Table 6. ‘Social value’ factor layer mean value matrix and weight.**

| **Matrix 4** | **Native Plant Ornamental Characteristics** | **Cultural Symbol Connotation** | **Conservation and Utilization of Old and Valuable Trees** | **Stayability** | **Completeness of Supporting Facilities** | **Safety** | **Landscape comfort** | **weighted value** |
| --- | --- | --- | --- | --- | --- | --- | --- | --- |
| Native Plant Ornamental Characteristics | 1.00 | 3.00 | 2.00 | 5.00 | 0.50 | 0.25 | 4.00 | 0.15 |
| Cultural Symbol Connotation | 0.33 | 1.00 | 0.50 | 3.00 | 0.25 | 0.20 | 2.00 | 0.07 |
| Conservation and Utilization of Old and Valuable Trees | 0.50 | 2.00 | 1.00 | 4.00 | 0.33 | 0.25 | 3.00 | 0.11 |
| Stayability | 0.20 | 0.33 | 0.25 | 1.00 | 0.17 | 0.14 | 0.50 | 0.03 |
| Completeness of Supporting Facilities | 2.00 | 4.00 | 3.00 | 6.00 | 1.00 | 0.50 | 4.00 | 0.23 |
| Safety | 4.00 | 5.00 | 4.00 | 7.00 | 2.00 | 1.00 | 6.00 | 0.36 |
| Landscape Comfort | 0.25 | 0.50 | 0.33 | 2.00 | 0.25 | 0.17 | 1.00 | 0.05 |

In this matrix, *λmax*=7.242, *C.I.*=0.040, *R.I.*=1.360, *C.R.*=0.030 was <0.1, and the consistency test was passed.

**Appendice 2 SBE Beauty Evaluation Questionnaire**

Dear Participants:

Greetings! Thank you for your participation in this plantscape evaluation questionnaire. I am a PhD student in Landscape Architecture, and the topic of this research focuses on the comprehensive evaluation of plantscapes in different urban green space habitats. To this end, this survey adopts the Scenic Beauty Estimation (SBE) method, which aims to collect public subjective aesthetic evaluation data of different plantscape units for subsequent scientific analysis and research.

Rating Description:

Please rate each of the botanical landscape photos below based on your personal intuition. The scoring criteria are as follows:

1 point: extremely unattractive

2 points: unsightly

3 points: Less aesthetically pleasing

4 points: average

5 points: more beautiful

6 points: beautiful

7 points: extremely beautiful

Please make your evaluation based on your first impression, no need to think too much, and choose only one score for each photo.

Example of scoring page:

GY1 Please select your rating (1-7)

GY2 Please select your rating (1-7)

...

QY7 Please select your rating (1-7)

QY8 Please select your rating (1-7)

A gentle reminder:

This survey is for academic research only and all your information will be kept strictly confidential.Please complete the evaluation independently in a quiet, distraction-free environment. Thank you for your serious participation!


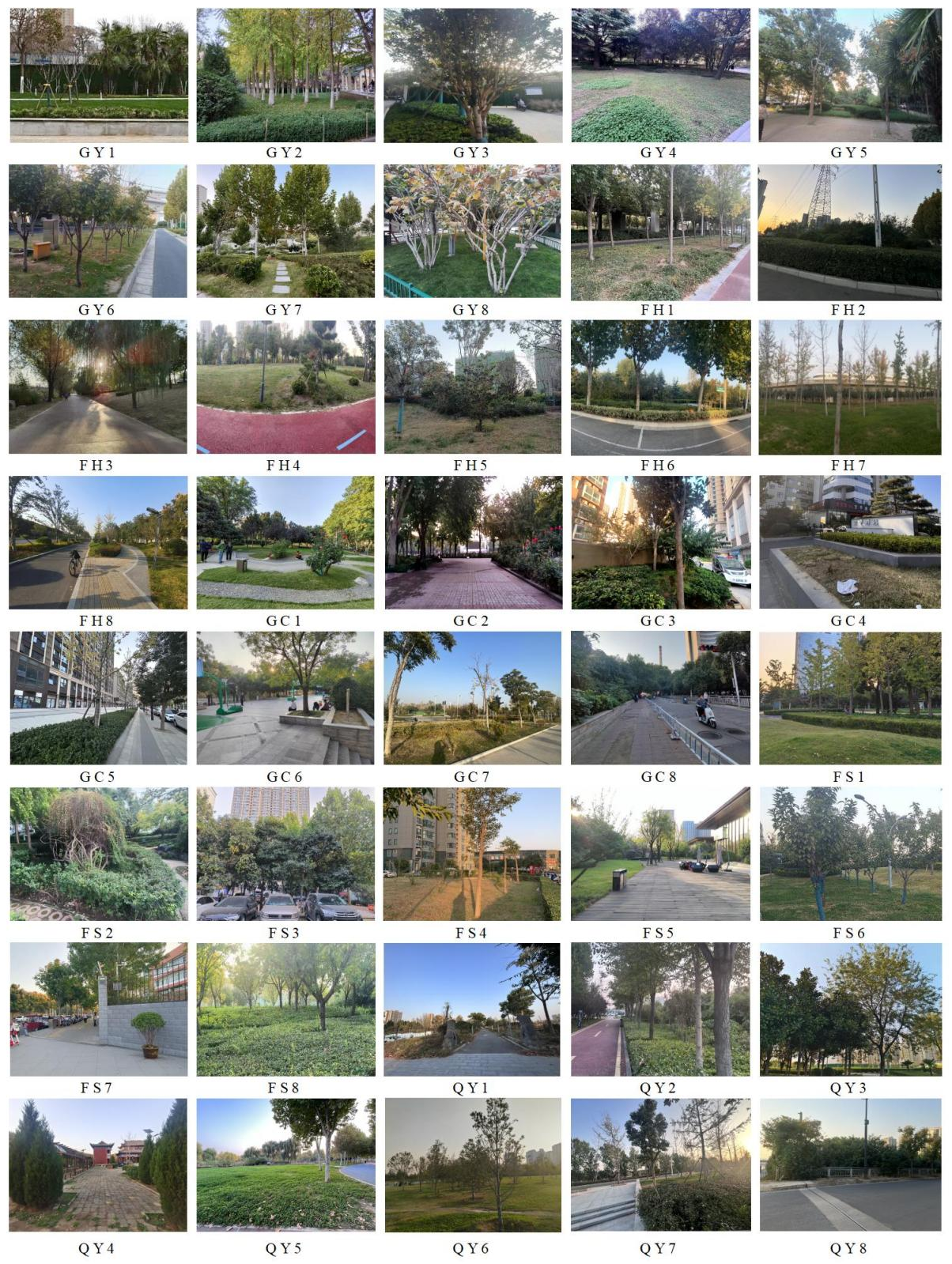

Supplement: S1 File — S1 Table. (DOCX). S2 File. (XLSX). S3 All the ethical parameters for human involvement in this study (PDF). (ZIP) [file pone.0329119.s001.zip › Appendices.docx]
